# Impulsive Approach Tendencies towards Physical Activity and Sedentary Behaviors, but Not Reflective Intentions, Prospectively Predict Non-Exercise Activity Thermogenesis

**DOI:** 10.1371/journal.pone.0115238

**Published:** 2014-12-19

**Authors:** Boris Cheval, Philippe Sarrazin, Luc Pelletier

**Affiliations:** 1 Univ. Grenoble Alpes, Lab. Sport et Environnement Social (SENS), F-38000, Grenoble, France; 2 School of Psychology, University of Ottawa, Ottawa, Canada; 3 Faculty of Psychology and Educational Sciences, University of Geneva, Geneva, Switzerland; West Virginia University School of Medicine, United States of America

## Abstract

Understanding the determinants of non-exercise activity thermogenesis (NEAT) is crucial, given its extensive health benefits. Some scholars have assumed that a proneness to react differently to environmental cues promoting sedentary *versus* active behaviors could be responsible for inter-individual differences in NEAT. In line with this reflection and grounded on the Reflective-Impulsive Model, we test the assumption that impulsive processes related to sedentary and physical activity behaviors can prospectively predict NEAT, operationalized as spontaneous effort exerted to maintain low intensity muscle contractions within the release phases of an intermittent maximal isometric contraction task. Participants (n = 91) completed a questionnaire assessing their intentions to adopt physical activity behaviors and a manikin task to assess impulsive approach tendencies towards physical activity behaviors (IAPA) and sedentary behaviors (IASB). Participants were then instructed to perform a maximal handgrip strength task and an intermittent maximal isometric contraction task. As hypothesized, multilevel regression analyses revealed that spontaneous effort was (a) positively predicted by IAPA, (b) negatively predicted by IASB, and (c) was not predicted by physical activity intentions, after controlling for some confounding variables such as age, sex, usual PA level and average force provided during the maximal-contraction phases of the task. These effects remained constant throughout all the phases of the task. This study demonstrated that impulsive processes may play a unique role in predicting spontaneous physical activity behaviors. Theoretically, this finding reinforces the utility of a motivational approach based on dual-process models to explain inter-individual differences in NEAT. Implications for health behavior theories and behavior change interventions are outlined.

## Introduction

Physical inactivity has been identified as one of the major risk factors for global mortality, causing an estimated 3.2 million deaths in the world and 32.1 million disability-adjusted life years [1]. Increasing Physical Activity (PA) is therefore one of the public health priorities. PA-related thermogenesis can be decomposed into volitional exercise thermogenesis (VET) and non-exercise activity thermogenesis (NEAT) [2], [3]. The former represents purposeful/deliberative PA, such as sport or scheduled PA, whereas the latter represents “physical activities other than volitional exercise, such as the activities of daily living, fidgeting, spontaneous muscle contraction, and maintaining posture when not recumbent” [2]. Although NEAT consists mainly of low-intensity behaviors, it accounts for substantial energy expenditure [2] and plays an important role in metabolic and cardiovascular health [4], [5]. Accordingly, understanding the determinants of NEAT is crucial.

Genetic, biological, and environmental determinants have been speculated [3], [6]. Some scholars [3] have assumed that inter-individual differences in NEAT are related to different responses to the environmental cues that promote sedentary *versus* active behaviors. This study is in line with this reflection. Grounded on the Reflective-Impulsive Model (RIM) [7] we investigated whether impulsive approach tendencies towards PA and sedentary behaviors (SB) are helpful in understanding inter-individual differences in NEAT.

### The Reflective-Impulsive Model

The RIM postulates the presence of two separate systems of information processing: the *reflective* and the *impulsive*. The reflective system is slow, effortful, and based on complex executive functions. Personal standards, reasoned evaluations of pros and cons, and action plans reside in the reflective system. Typically seen as reasoned, conscious and intentional, these plans and decisions activate proper behavioral schemata (e.g., “I intend to walk three times a week for at least 30 minutes each time to improve my health”). By contrast, the impulsive system is quick and based on automatic associative processes that the person has acquired. For example, through the repeated experience of a PA behavior (e.g., climbing stairs), an associative cluster may be formed that links (a) the concept of stairs with (b) the negative (or positive) affect felt during behavioral execution, and (c) the behavioral schema that has led to the affect (e.g., approach or avoidance towards stairs). Once established within the impulsive system, associative clusters can be reactivated quickly by mere perceptual input (e.g., seeing the stairs) and further guide attention and information processing. They are associated with largely automatic approach-avoidance tendencies towards the object, which prepare the organism to execute related behavioral schemata [8]. As a result, positive associations should increase a person's inclination to carry out the behavior and negative ones should decrease it. Such associative processes are generally assumed to be independent of conscious awareness and to operate in an effortless manner [7].

The RIM postulates that both impulsive and reflective processes may determine behaviors, albeit to different degrees depending on the level of controllability of the behavior [9]. Specifically, the reflective system should underlie the regulation of deliberate, largely controlled forms of behavior, whereas the impulsive system should underlie the regulation of impulsive, largely automatic forms of behavior. For example, some PA-like exercise and sport carried out in highly organized and structured settings are presumably guided predominantly by reflective processes. By contrast, NEAT is presumably more influenced by impulsive processes. Indeed, if people attempted to make all of their decisions to involve themselves in such activities through reflective thought, they would quickly exhaust their limited resources for self-regulation [10].

While the RIM has proven to be useful to predict behaviors relating to employee safety [11], voting [12] and health (see [8], [13] for reviews), only two studies have assessed impulsive processes to prospectively predict PA behaviors [10], [14]. The Conroy et al. study revealed that impulsive processes towards PA accounted for variability in walking behavior over a 7-day pedometer assessed monitoring period above and beyond reflective processes (e.g., behavioral intentions). Adopting a broader perspective by including impulsive processes related to SB likely to hinder PA behavior, the accelerometer assessed Cheval et al. study revealed that impulsive approach tendencies towards PA and towards sedentary behaviors predicted respectively positively and negatively moderate to vigorous PA (MVPA) over one week, over and above PA intentions. However, in these studies the use of daily step counts and MVPA as dependent variables did not allow VET and NEAT to be differentiated. Indeed, the pedometer and accelerometer assessment of PA over a one week period included activities that vary with respect to the level of spontaneity *versus* deliberation (e.g., a spontaneous walk to move from one place to another *versus* a scheduled walk every Sunday morning). Accordingly, further tests — examining spontaneous PA behaviors more accurately — are crucial in order to make the case that impulsive precursors constitute important predictors of such behaviors.

### The present study

The purpose of the current study was to examine whether impulsive processes can prospectively predict NEAT. This was operationalized as spontaneous effort provided to maintain low intensity muscle contractions (SLIMC) during the relative release phases of an intermittent maximal isometric contraction (IMIC) task, during which the participants were told to maintain slight pressure on the handgrip. Impulsive approach tendencies towards PA (IAPA) and towards sedentary behaviors (IASB) were assessed using a manikin task based on work by De Houwer and colleagues [14], [15], a well-validated measure of impulsive approach-avoidance tendencies [16]. Intentions to be physically active were assessed to measure reflective precursors of PA behaviors. Based on the RIM and earlier studies, we hypothesized that SLIMC should be positively predicted by IAPA, negatively predicted by IASB, and not predicted by reflective PA intentions, after controlling for some confounding variables (i.e., age, sex, BMI, and usual PA level) likely to influence both the average level and growth of the spontaneous effort.

## Method

### Ethics statement

The University of Ottawa Institutional Review Board (IRB) for the Social Sciences approved this research and informed consent process (#06-12-26). In agreement with the Canadian Tri-Council Policy Statement (TCPS): Ethical Conduct for Research Involving Humans (http://www.pre.ethics.gc.ca/eng/policy-politique/initiatives/tcps2-eptc2/Default/) all participants were given written informed consent prior to participation, and received a written debriefing at the end of each study session. According to the TCPS, no parent/guardian's consent was required as all the participants were 16 years of age (http://www.hc-sc.gc.ca/sr-sr/advice-avis/reb-cer/consent/f-eng.php).

### Participants and procedure

To be included in the study, participants had to be free of any medical conditions that would prohibit PA without supervision, and not currently be receiving treatment for a psychiatric disorder. Participants with diabetes, history of alcohol, or drug use were also excluded from the study. Ninety-one University of Ottawa undergraduate students (76 women and 15 men; *M_age_*  = 18.93, *SD*  = 2.69) who met the eligibility criteria participated in exchange for course credits. They first completed a questionnaire assessing their usual level of PA, their intentions to such behaviors over the next week, and provided some demographic information (i.e., age, sex). Participants were then seated in front of a computer in order to complete the manikin task. Immediately afterwards, they were asked to carry out two handgrip tasks: a maximal handgrip strength task and an IMIC task.

### Measures

#### Usual level of PA

A self-administered short version of the International Physical Activity Questionnaire (IPAQ) [17], slightly modified to control for the “usual” level of PA, was used. Specifically, questions focused on the time spent in moderate and vigorous activities of at least 10 minutes (at a time) over a “typical week”. Standard IPAQ descriptions were used to define moderate (“activities that require moderate physical effort and make you breathe somewhat harder than normal”) and vigorous (“activities that that require hard physical effort and make you breathe much harder than normal”) activities. Participants indicated the number of days they engaged in these activities and for how long on each occasion. Multiplying frequency and usual duration gave the average amount of time spent in exercise activities per week.

#### Physical activity intentions

Two items assessed participants' willingness and intention to engage in the recommended amount of PA over the next week during free time (e.g., over the next seven days, I intend to participate in at least 30 minutes of moderate-to vigorous-intensity physical activity, 5 times a week, during my free time) using a 7-point Likert scale (1 = strongly disagree, 7 = strongly agree). These items correlated strongly (r = .87) and were combined into a single score.

#### Impulsive approach-avoidance tendencies towards PA and SB

**Figure 1:**
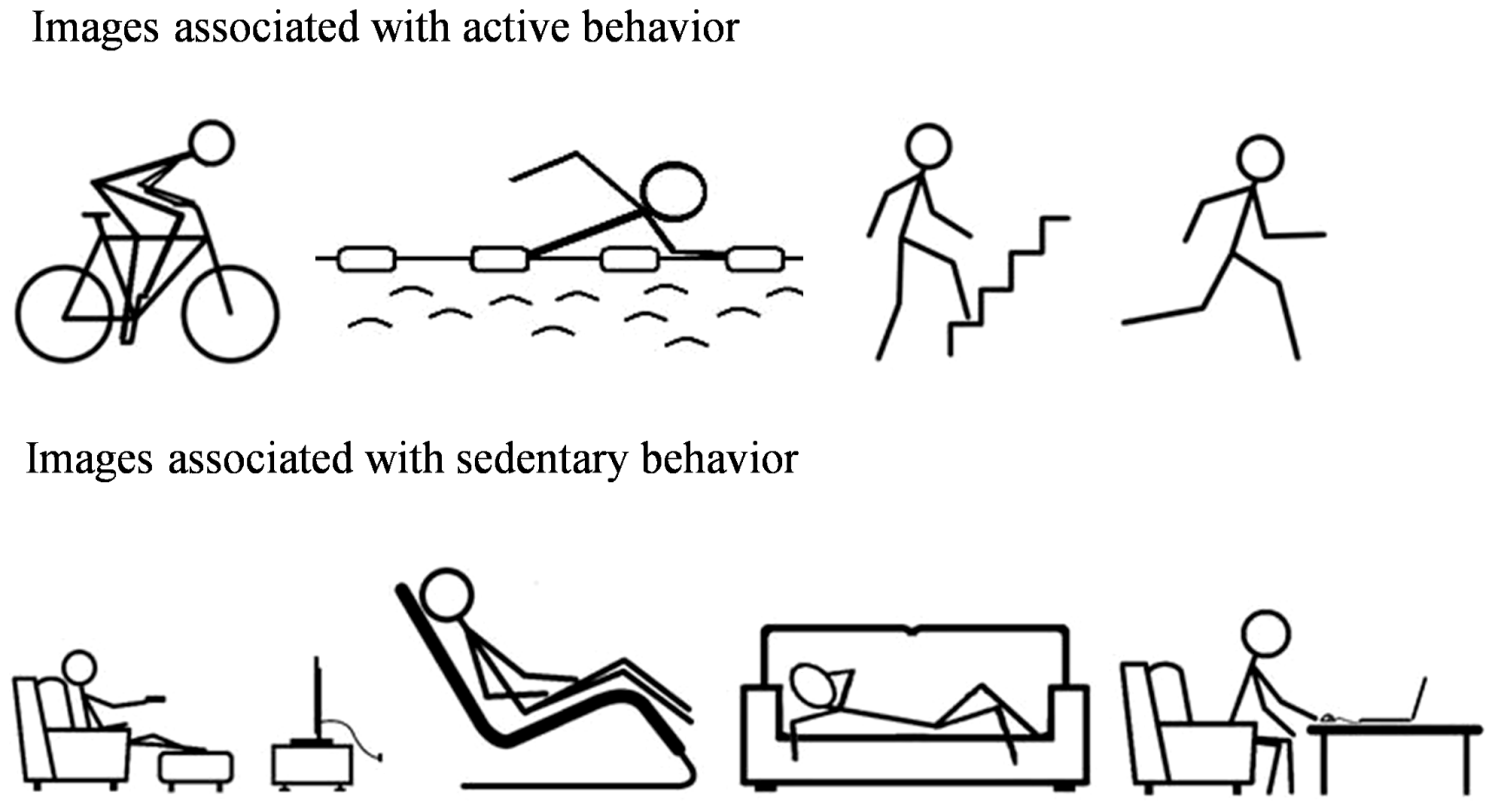
Sample of images used in the manikin task.

**Figure 2:**
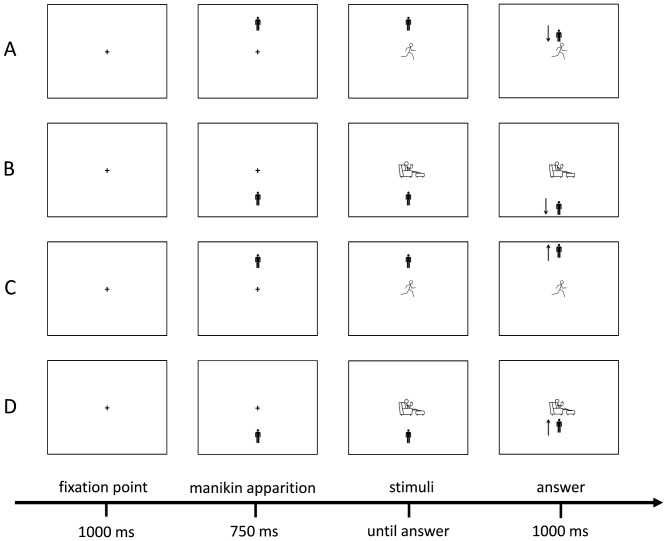
Illustration of the manikin task. A. represents a trial in which participants were instructed to approach physical activity (PA) images (32 trials). B. represents a trial in which participants were instructed to avoid sedentary behavior (SB) images (32 trials). C. represents a trial in which participants were instructed to avoid PA images (32 trials). D. represents a trial in which participants were instructed to approach SB images (32 trials). The arrows down and up indicate the keyboard key on which the participant had to press for the right answer.

IAPA and IASB were measured using the same manikin task as in the Cheval et al. study [14]. Using Eprime software, participants were asked to move a manikin – i.e., a schematic image of a human figure – upwards or downwards by repeatedly pressing the “8” or “2” keys respectively. Each trial started with a fixation cross in the middle of the screen. Participants had to press the “5” key and keep it pressed until they began to move the manikin. The manikin could appear in either the upper or the lower half of the screen with the same probability. After the appearance of the manikin, an image of PA or SB (pictograms representing either “movement and active lifestyle” or “rest and sedentary lifestyle”; see Fig. 1 for examples) was presented at the center of the screen. Depending on the condition, participants were asked to move the manikin as quickly and as accurately as possible “toward” a PA image and “away” from an SB image, or vice versa. If an incorrect response was made, error feedback appeared on the screen. Five hundred milliseconds (ms) after the third key press, the screen was cleared for 1000 ms before the start of the next trial. The reaction time (RT) between the appearance of the image and the first key press was used in the analyses. Participants completed two blocks of trials, each consisting of 12 practice trials and 64 test trials (i.e., each of the 16 images appeared twice in the top and twice in the bottom of the screen). In one block, participants were instructed to approach PA images and to avoid SB images, and in the other block, they were instructed to do the opposite. The order of the blocks was counterbalanced across participants (see Fig. 2 for illustration).

Before analyzing the data we excluded incorrect responses (4.6%) as well as responses below 150 ms (<0.001%) and above 1,500 ms (0.08%) as recommended by [16]. Results are presented with the median RT. Participants' impulsive approach tendencies toward PA and SB were calculated by subtracting the median avoidance RT from the median approach RT toward PA and SB images respectively. A positive score always indicates a tendency to approach rather than avoid the behavior. Past studies [15], [16], [18] showed the validity and reliability of manikin tasks to assess impulsive approach-avoidance tendencies. In this study, reliability was good (α  = .75 and.83 for PA and SB, respectively).

#### Handgrip task

Isometric grip force was measured using a handgrip dynamometer (Vernier Software & Technology; accuracy, 0.6 N). Handgrip performance has already shown to be correlated with electromyography activity [19], [20], and therefore appears as a valid method to assess muscle activity. This device allows the pressure induced by the force exerted on the grip to be recorded continuously at a 24-Hz frequency. Participants were seated with the dynamometer in their dominant hand, wrist neutral, elbow flexed to 90 degrees, and shoulder adducted and neutrally rotated. After a period of familiarization with the tool, the participants' maximal handgrip strength was established by asking them to squeeze the handgrip as strongly as possible for 15 seconds (s). Then, after a 5-minute rest period, they were instructed to perform the IMIC task. This consisted of twenty 5 s maximal contractions with 10 s relative releases in-between the maximal contractions. Specifically, participants were instructed to squeeze the handgrip as strongly as possible on a first auditory cue, then on a second auditory cue, to recover by relaxing the force of the squeeze but still maintain a slight pressure and to repeat this procedure twenty times. The maximal-contraction phases were designed both to prime participants with the PA context and to reduce their attention during the relative release phases. The strength involved during the relative release phases corresponds to SLIMC that individuals have to engage in to maintain certain postures in everyday life, one of the components of NEAT [2]. The data were averaged over one second, so that each participant had 190 timed measurements of spontaneous muscle contraction force. In order to control for differences in strength between participants, the SLIMC provided by participants was expressed in percentage of their maximal handgrip strength. This score was used as the dependent variable in the analyses.

### Data analysis

Data were analyzed using hierarchical linear modeling (HLM), given that the present study involved a hierarchically structured data set where the SLIMC are nested within individual. HLM is a flexible approach that can be applied to evaluate inter-individual differences in intra-individual changes over time. That is, HLM separates inter-individual variance from intra-individual, so that each participant has his/her own curve [21], [22]. HLM accounts for the shared variance by multiple observations within the same participant (i.e., non-independence). In other words, given that the sampling variance is taken into account, the true rate of change and the true status at each point in time can be modeled. Thus, the parameter estimates generated from HLM (particularly the standard errors) are less biased than those generated from generalized linear models.

Two models were examined. *Model 1* was an unconditional growth model that estimated the average, as well as the individual differences in intercept and growth trajectory. At level 1, time (i.e., linear development) and time squared (i.e., quadratic development) were entered as predictors to estimate the average intercept, the average linear and quadratic growth trajectories. The time was centered at the first of the 190 time measurements. Thus, the intercept should be interpreted as the level of isometric grip force at the beginning of the relative release phases. The random effect of both the intercept and the linear slopes were included in the model. *Model 2* estimated the effect of impulsive processes (i.e., IAPA and IASB; Level 2 predictors) on an average level of SLIMC as well as the cross-level moderating role of these impulsive processes on both the linear and the quadratic growth trajectories of SLIMC. Only significant interactions were retained in order to permit more powerful tests of the remaining terms and to simplify the model as much as possible [23]. In addition, the effects of the reflective processes (i.e., PA intentions) and the control variables (i.e., age, sex, usual PA level, and mean force provided during the maximal-contraction phases of the IMIC task) on the spontaneous handgrip effort were controlled for. All predictors were centered at sample mean (i.e., grand mean centering) and the dichotomous variables were dummy coded [21].

## Results

### Preliminary analysis

Means, standard deviations, and bivariate correlations are presented in Table 1.

**Table 1:** Descriptive statistics and intercorrelations between variables (N = 91).

	Variables	Mean	SD	1	2	3	4	5	6	7
1	PA intentions	4.9	1.21	_						
2	IAPA	115.31	124.36	0.09	_					
3	IASB	−4.8	150.77	−0.13	−0.42**	_				
4	SLIMC^1^ (% Fmax)	5.36	2.88	0.04	0.30**	−0.30**	_			
5	MCP^1^ (% Fmax)	48.03	10.9	0.28**	0.11	−0.01	−0.12	_		
6	Age	18.93	2.69	−0.15	−0.06	−0.12	0.28**	0.08	_	
7	Sex^2^, women number (%)	76 (83.5)	-	−0.12	−0.13	0.19	0.19	−0.18	−0.13	_

Note. PA  =  physical activity; IAPA =  Impulsive approach tendency towards PA; IASB  =  Impulsive approach tendency towards sedentary; SLIMC  =  spontaneous effort provided to maintain low intensity muscle contractions; MCP  =  maximal-contraction phases.

1 For each participant a mean score of SLIMC and MCP through all the phases of the task was calculated.

2 women = −0.5, men = 0.5.

* *p*<.05.

** *p*<.01.

### Spontaneous handgrip effort trajectory

Results of Model 1 (see Table 2) revealed significant negative linear effect and positive quadratic effect of time on SLIMC. Specifically, SLIMC decreased between 1 and 146 s, then remained constant until 175 s before increasing up to 190 s.

**Table 2:** Multilevel regression models to examine the effect of reflective and impulsive precursors on SLIMC during the relative release phases of a handgrip task.

Predictors	Model 1		Model 2	
	*b*	*SE*	*b*	*SE*
**Fixed Effects**				
Intercept	8.86	0.46***	9.96	0.53***
Time	−0.06	0.003***	−0.64	0.003***
Time squared	2.00E-04	1.4e-05***	2.01E-04	1.44e-05***
Sex^1^			3.28	1.07**
Age			0.43	0.15***
Usual PA behavior			0.005	0.002*
Mean Force during the MCP			−6.76	3.72
PA Intentions			−0.17	0.29
IAPA			0.009	0.003*
IASB			−0.006	0.003*
**Random Effects**				
Intercept	18.2	0.279***	12.67	1.96***
Time	3.22E-04	5.5e-05***	3.18E-04	5.40e-05***
Error	25.85	0.279***	25.85	0.279***
-2 Log likelihood	105941.125		105909.282	

Note. SLIMC  =  spontaneous effort provided to maintain low intensity muscle contractions; ^1^women = −0.5, men = 0.5; MCP  =  maximal-contraction phases; PA =  Physical Activity; IAPA =  Impulsive approach tendency towards PA; IASB  =  Impulsive approach tendency towards sedentary.

*p<.05.

**p<.01.

***p<.001.

### Effects of IAPA and IASB on spontaneous handgrip effort

**Figure 3:**
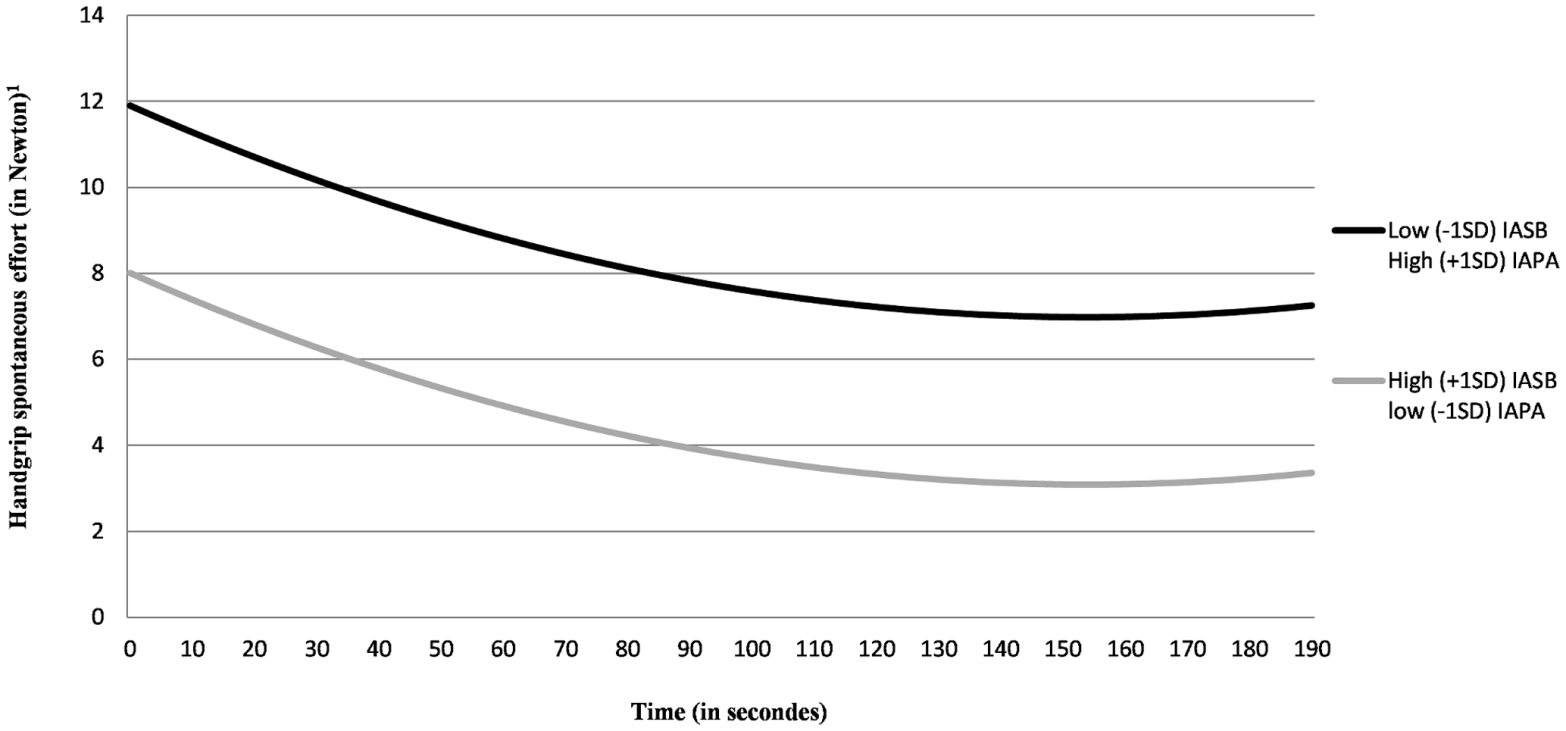
SLIMC trajectory during the release phases of the PA handgrip task depending on the impulsive approach tendencies towards PA and SB.

Results of Model 2 (see Table 2) revealed that SLIMC at the beginning of the task were positively related to sex (higher for men), age, and usual PA behavior. In line with our expectations, SLIMC at the beginning of the handgrip task were not predicted by PA intentions, but were predicted by IAPA and IASB (positively and negatively, respectively). Moreover, these two impulsive processes did not significantly moderate the linear (*p*s>.24) and quadratic (*p*s>.37) SLIMC trajectories. Given that IAPA and IASB moderately negatively correlated (*r* = −.42, *p*<.01), we decided to plot the SLIMC trajectory for participants with high IAPA and low IASB (i.e., with an impulsive system predisposing to active behavior) and for those with low IAPA and high IASB (i.e., with an impulsive system predisposing to inactive behavior). As depicted in Fig. 3, participants with an impulsive system predisposing to active behavior had a higher level of SLIMC of 3.89% of their maximum strength, as compared to those with an impulsive system predisposing to inactive behavior (8.33% *versus* 4.44% of their maximum strength on average). In other words, the more participants have an impulsive system predisposing to active behavior the more SLIMC they invested in the task. In this model, the variables under consideration explained 30.4% of the inter-individual variances in SLIMC, of which 14.5% was explained only by IAPA and IASB.

## Discussion

Promoting regular PA is one of the public health priorities given its extensive health benefits [24], but low participation rates still remain [25]. PA thermogenesis can be decomposed in VET and NEAT [2]. Although NEAT consists mainly of low intensity behaviors, they account for substantial energy expenditure, up to 2000 kcal per day [26] and play an important role in metabolic and cardiovascular health [4], [5]. Understanding the determinants of these kinds of behaviors is therefore crucial. Some scholars [3] have assumed that a proneness to react differently to environmental cues promoting sedentary *versus* active behaviors could be responsible for inter-individual differences in NEAT. In line with this reflection and grounded on the RIM, the current study tested the assumption that impulsive processes related to PA and SB can prospectively predict some components of NEAT, such as SLIMC, engaged in during the relative release phases of an IMIC task. While such effort appears low in intensity (5.36% of the maximum handgrip strength on average; see Table 1), they correspond to the effort individuals have to make in everyday life when they maintain certain postures. Inter-individual differences in posture allocation are not trivial and can account for a variability of up to 352 kcal per day [2].

We hypothesized that SLIMC should be positively predicted by IAPA, negatively predicted by IASB, and not predicted by reflective PA intentions, after controlling for some confounding variables such as age, sex, usual PA level and average force provided during the maximal-contraction phases of the IMIC task. The results of our study corroborated these hypotheses. Moreover, the relationship between the two impulsive processes and SLIMC remained constant throughout all the phases of the IMIC task, and after controlling for some confounding variables. Together, IAPA and IASB led to a sizable 14.5% increase in explained SLIMC variance. This result is in agreement with those from the two previous studies showing that impulsive processes prospectively predicted PA behaviors [10], [14]. However, in these studies, the use of daily step counts and MVPA as dependent variables did not allow different types of PA behavior, such as NEAT *versus* VET to be differentiated. Accordingly, we believe the present study is the first to demonstrate that impulsive precursors of behavior can prospectively predict SLIMC, one of the components of NEAT [2]. In addition, this study replicated the negative effect of IASB on PA behaviors found in an earlier study [14] and confirms the importance of not only examining impulsive processes directly related to the construct of interest (i.e., PA), but also examining impulsive processes related to behaviors that could hinder PA behavior implementation, such as sedentary behaviors.

It appears quite remarkable that reaction-time-based tasks indicating strong predispositions to approach (rather than to avoid) PA and/or activities that are at odds with PA, are positively and negatively related respectively, to SLIMC. Such tasks provide indirect measures of associative structures within the impulsive system [27]. These associative clusters connecting a concept, an affect, and a behavioral schema, prepare the organism to evaluate and respond to the environment quickly in accordance with previous learning experiences [7]. As a result, the impulsive systems of individuals with positive association towards PA and/or negative associations towards SB predispose them to exert more effort on SLIMC when opportunities to engage in such behaviors arise, compared to participants having impulsive systems with negative association towards PA and/or positive association towards SB.

A practical implication of this result is that a comprehensive consideration of the impulsive processes involved in NEAT should be required when developing interventions to increase these kinds of PA behaviors. Specifically, the potential for information-based intervention to change NEAT is fundamentally limited, because these kinds of behaviors do not appear to be driven by reflective precursors of actions, such as intentions. Accordingly, interventions designed to directly influence automatic associative processes should be more beneficial in promoting spontaneous PA than reflective ones. For example, interventions altering or creating new associations such as evaluative conditioning [28] or retraining automatic action tendencies [29] should foster NEAT. Based on the present findings, these procedures could target either strengthening IAPA or reducing IASB, given that these impulsive processes were, respectively, positively and negatively related to NEAT.

Although the present study was designed to address some of the limitations observed in previous studies, it has a few limitations of its own. First, our study used a college student population. It is thus unclear whether conclusions could generalize to a population more heterogeneous across age range. Second, we assessed only the spontaneous effort exerted to maintain posture in a laboratory-based measure. We recognize that our ability to measure NEAT in a free-living people was limited in this study. Indeed, NEAT are broader and include ambulation and fidgeting as well as body posture [26]. Nonetheless the current study showed for the first time that impulsive approach-avoidances tendencies towards PA and SB predict inter-individual differences in spontaneous handgrip effort. Future studies should adopt the Levine et al. [26] protocol for assessing the impact of IAPA and IASB on the different NEAT components in free-living humans.

In conclusion, this study demonstrated that impulsive processes play a unique role in predicting spontaneous PA behaviors. It also confirms that impulsive processes can either predispose (i.e., IAPA) or compromise (i.e., IASB) such behaviors. These findings reinforce the utility of a motivational approach based on dual-process models to explain inter-individual differences in NEAT. Finally, a clear implication of the present findings is that targeting reflective processes may be ineffective in promoting NEAT, while in contrast, targeting impulsive processes seems particularly promising. We hope that these results will stimulate more research on the mechanisms involved in the regulation of such important behaviors for our health, and will stimulate initiatives to develop more comprehensive interventions targeting both impulsive and reflective mechanisms. Such dual-process-based interventions appear to hold great promise in improving public health.
